# Modified GAN Augmentation Algorithms for the MRI-Classification of Myocardial Scar Tissue in Ischemic Cardiomyopathy

**DOI:** 10.3389/fcvm.2021.726943

**Published:** 2021-09-13

**Authors:** Umesh C. Sharma, Kanhao Zhao, Kyle Mentkowski, Swati D. Sonkawade, Badri Karthikeyan, Jennifer K. Lang, Leslie Ying

**Affiliations:** ^1^Department of Medicine, Division of Cardiology, Jacobs School of Medicine and Biomedical Sciences, Buffalo, NY, United States; ^2^Department of Pharmacology and Toxicology, University at Buffalo, Buffalo, NY, United States; ^3^Department of Biomedical Engineering, University at Buffalo, Buffalo, NY, United States; ^4^Veterans Affairs Western New York Healthcare System, Buffalo, NY, United States

**Keywords:** cardiac MRI, generative adversarial networks, data augmentation, myocardial scarring, deep learning

## Abstract

Contrast-enhanced cardiac magnetic resonance imaging (MRI) is routinely used to determine myocardial scar burden and make therapeutic decisions for coronary revascularization. Currently, there are no optimized deep-learning algorithms for the automated classification of scarred vs. normal myocardium. We report a modified Generative Adversarial Network (GAN) augmentation method to improve the binary classification of myocardial scar using both pre-clinical and clinical approaches. For the initial training of the MobileNetV2 platform, we used the images generated from a high-field (9.4T) cardiac MRI of a mouse model of acute myocardial infarction (MI). Once the system showed 100% accuracy for the classification of acute MI in mice, we tested the translational significance of this approach in 91 patients with an ischemic myocardial scar, and 31 control subjects without evidence of myocardial scarring. To obtain a comparable augmentation dataset, we rotated scar images 8-times and control images 72-times, generating a total of 6,684 scar images and 7,451 control images. In humans, the use of Progressive Growing GAN (PGGAN)-based augmentation showed 93% classification accuracy, which is far superior to conventional automated modules. The use of other attention modules in our CNN further improved the classification accuracy by up to 5%. These data are of high translational significance and warrant larger multicenter studies in the future to validate the clinical implications.

## Introduction

Acute myocardial infarction (MI), commonly known as a heart attack, is an unpredictable complication of coronary artery disease (CAD). The location, size, density and heterogeneity of myocardial scarring provides both diagnostic and prognostic information for patient management. Such information is critical to manage patients at risk for heart failure (HF) and lethal cardiac arrhythmias (1, 2). HF and cardiac arrhythmias usually result from diseased myocardium and electrically unstable scars (3–5). Therefore, myocardial scar classification using emerging data augmentation methods is of great clinical significance.

Deep learning and artificial intelligence are rapidly gaining importance in the field of medical imaging. The development of newer generation cardiac MRI scanners with a higher signal-to-noise ratio and better edge definition has enabled us to better characterize myocardial scar tissue. However, we still lack smart decision-making tools to accurately classify the “*scar tissue*” in an objective and reproducible manner. Fortunately, there are major enhancements in the Graphics Processing Unit (GPU) development that enable us to train a large dataset in a relatively short time span.

A promising approach to improve the accuracy and consistency of myocardial scar detection lies in artificial intelligence – the use of a machine to perform deep cognitive analysis based on data input. The availability of such platforms has the potential to improve clinical workflow, enhance diagnostic accuracy, and offer options for early interventions. Furthermore, integrating preclinical and clinical analytical algorithms will allow us to directly examine the clinical implication of scar tissue after acute MI in humans, which has the potential to have an immediate impact on patient management.

Since its inception in 2012, the ImageNet classification platform with convolutional neural networks (CNNs) has been developed as the most efficient data analytical platform (6). Well-designed CNN models, such as VGG, Inception v3, and Resnet 50 have shown exceptional performance for image classification, and now deep learning applications are frequently used in medical image analysis (7–9). To enhance accuracy, multiple applications including feature detection, segmentation, classification, and image reconstruction are being integrated into various data platforms (10, 11).

In this translational study, we generated the first proof-of-concept data in a pre-clinical model of acute MI induced by ligating the left anterior descending coronary artery. The mice then underwent contrast-induced cardiac MRI and confirmatory histology analysis of the infarct and remote regions. After preclinical testing, we studied over 7,000 augmented images generated from patients with a history of CAD and known myocardial scar development.

## Methods

### Mouse Model of Acute MI

All preclinical procedures and protocols conformed to institutional guidelines for the care and use of animals in research and were reviewed and approved by the University at Buffalo Institutional Animal Care and Use Committee (IACUC). Acute MI was induced in mice (age 14–15 weeks, C57B1/6 background) by using our study protocol described previously (12–14). Mice underwent permanent ligation of the left anterior descending (LAD) coronary artery producing an infarct in the anterior/anteroseptal walls of the LV. Concisely, mice were anesthetized with ketamine (1 mg/kg intramuscular) and xylazine (5 mg/kg subcutaneous) and were intubated to undergo a ligation procedure (9-0 nylon) of the LAD. Our laboratory performs AMI experiments on a routine basis with 70–80% post-MI survival. We studied 12 survivor mice (6 MI and 6 controls) for 2 weeks. On day 14, mice underwent cardiac MRI with gadolinium contrast infusion. To minimize pain and distress, all studies were performed on anesthetized (1.5% of isoflurane) animals. Upon completion of cardiac MRI, mice were sacrificed using the CO2 euthanasia protocol approved by the IACUC. The euthanasia procedure conformed to the guidelines from the Panel on Euthanasia of the American Veterinary Medical Association.

### Myocardial Histology

Myocardial histology was performed to provide a gold-standard (tissue) validation of myocardial infarction. Since Human subjects are not required to undergo cardiac biopsy for tissue validation of myocardial scar, pre-clinical studies were performed for the conclusive evidence of myocardial infarction, along with cross-validation with cardiac MRI in mice.

To visualize the extent of MI in mice, an Evans Blue/tetrazolium chloride (TTC) method was employed. For the TTC assays, 0.5 mL of a 2% Evans blue solution (Sigma) devoid of bubbles was slowly perfused, turning the heart blue except for the risk regions. The heart was then removed, rinsed with KCl and PBS, and chilled at -20°C for 5 min prior to sectioning the LV into 7–8 transverse rings of 1 mm thickness using a heart slicer matrix (Zivic Instruments). Sections were subsequently incubated in a 1% TTC solution (Sigma) in a 37°C incubator for 15 min until a red stain developed to assess infarct size. The sections were placed between two clamped pieces of plexiglass with a 2 mm spacer and digital images were taken of both sides of each slice as described previously by our group (15). The total area and left ventricular (LV) area were calculated using Fiji. Using a color thresholding technique, we classified the blue regions of the heart as viable and the bright red as the risk regions.

Hematoxylin and eosin (H&E)-stained myocardial tissue sections were used to examine the infarct zone using the whole heart tissue, covering both ventricles and interventricular septum. Whole heart images were obtained from Leica Aperio VERSA whole slide imaging System at 63 × magnifications (Multispectral Imaging suite, University at Buffalo). The extent of total myocardial fibrosis was visualized by trichrome staining (Thermo Scientific™ Richard-Allan Scientific™ Masson Trichrome Kit/22110648). The total myocardial area and the area of positive staining for fibrosis were quantified using color deconvolution algorithms as described previously (16).

### Preclinical Cardiac MRI

Based on our study protocol explained previously (14), we used a 20 cm diameter horizontal-bore 9.4 Tesla magnet (Biospec 94/20 USR, Bruker Biospin) equipped with a gradient coil supporting 440 mT/m gradient strength and 3,440 T/m/s maximum linear slew rate (BGA-12S HP; Bruker Biospin). A series of three orthogonal gradient echo (GRE) scout images of the heart were acquired. For the tagged images, we acquired ECG and respiration-gated axial views of the heart in cine mode with 2D SPAMM tagging (0.1 mm thickness; 0.5 mm grid distance) using a single-slice fast low-angle shot (FLASH) sequence with the following parameters: 2 ms Gaussian pulse for slice selection; 30 flip angle; TE/TR = 2.2/15 ms; 50 kHz readout bandwidth; fat suppression; 1 mm slice thickness; 30 × 30 mm^2^ field of view; 256 × 256 matrix; 8 averages; 8 cardiac movie frames. The scan time was between 10 and 15 min, depending on respiration and heart rate.

For late gadolinium enhancement (LGE), we discharged the syringe and acquired late contrast enhancement data at 20 min after the injection using an ECG and respiration-gated inversion-recovery T_1_-weighted FLASH sequence with the following parameters: 60° flip angle; TE/TR = 2.1/1200 ms; 65 kHz readout bandwidth; TI = 200 ms; 8 axial slices with 0.8 mm thickness and 0.2 mm gap; 25 × 25 mm^2^ field of view; 256 × 256 matrix; no averages. The scan time was between 3 and 5 min, depending on respiration and heart rate. Cardiac MRI images were taken both before and after the MI induction procedure (median time = 2 weeks), which were denoted as pre-MI and post-MI images, respectively, as described previously by our group (14). We selected 272 images from the MI group and 383 images from the normal control mice. After an initial quality review, 392 images were chosen as the training dataset.

### Contrast-Enhanced Cardiac MRI Protocol in Patients

Human experimental protocols were approved by an institutional review board (IRB) committee from University at Buffalo and all methods involving human/human data were performed in accordance with the relevant guidelines and regulations. Since the MRI database was accessed retrospectively and no direct patient contact was involved, the informed consent was waived by the IRB committee. Patient identifiers were securely processed using our existing Health Insurance Portability and Accountability Act (HIPAA) guidelines. A GE 1.5-T scanner with technical parameters recommended by the manufacturer was used. LGE sequence was obtained after intravenous (IV) gadolinium injection with an inversion recovery prepared T1 gradient echo and manually adapted inversion time. Typically, the images taken after 7–10 min after gadolinium injection were used for the current data analysis algorithms. Further details on cardiac MRI protocols were reported previously (17).

### Clinical MRI Dataset

We first obtained 1,447 images from 91 patients with a history of coronary artery disease. Most of these patients were referred for contrast-enhanced cardiac MRI after visualization of coronary artery disease on the invasive coronary angiogram (44% had a prior history of stent placement and 13% had previously undergone surgical revascularization). Patients underwent a comprehensive MRI protocol including gadolinium contrast injection. The presence of abnormal LGE signal after optimal inversion recovery in the contrast-enhanced MRI was considered as the presence of myocardial scar. Additionally, 313 MR images from 31 control subjects were used for comparison. The controls included age-matched subjects with identical myocardial function, but no evidence of myocardial scar. Only 660 images with abnormal LGE, and 207 control images passed the initial image quality review. The DCM image format was then transformed into JPG format using MicroDicom viewer before further processing of the datasets. Representative examples are shown in Supplementary Figure 1. The model was first tested with 206 images (103 randomly split from each category) to precisely evaluate the model classification accuracy. Next, 557 images from patients with ischemic scars and 104 control images were used for training. Since there were discrepancies between the number of MI and control images, we augmented the control data size to match the myocardial scar data size. To ensure a balanced comparative dataset, we adjusted the training sample size from both classes as demonstrated in data Supplementary Figure 2.

### MobileNetV2

MobileNet/MobileNetV1 was first proposed by a Google researcher team in 2017 (18). In this modified CNN model, a convolutional layer is replaced by a depthwise-separable convolution layer to reduce the parameters and speed-up the training process. The main refinement of MobileNetV2 is to improve the precision by the introduction of inverted residual blocks. The basic idea of residual blocks is derived from Resnet (9, 19). The inverted residual in MobileNetV2 reverses the residual block sequence in Resnet. Considering the accuracy and training speed, we used MobileNetV2 as the fundamental model in our experiment. To improve this model performance, *Finetune* (initialization by a pre-trained classification network, and then training for a different task) was used. Since pretrained parameters provide an excellent initiation point, *Finetuning* is widely employed in medical image analysis for a faster convergence of the model (10, 20, 21). The comparison of random initialization and pretrained parameter initialization is shown in Supplementary Figure 3.

### Attentional Units

To further enhance the MobileNetV2 classification accuracy, squeeze-and-excitation block (22) is considered as a channel attention (CA) unit to embed into MobileNetV2. The first layer in the squeeze-and-excitation block is a convolutional step. The remaining structure is similar to the residual block. First, a global average pooling is used to obtain individual channel information U. The key formulation is defined as S = σ(*g*(*z, W*)) = σ(*W*_2_δ(*W*_1_*z*)), where σ is a sigmoid activation and δ refers to a ReLU activation. These two activation layers learn non-linear interactions between the channels and generate a mask (S) of multiple channels to emphasize features. The output is the channel-wise multiplication from mask S and feature map U. Spatial attention (SA) is derived from convolution block attention module (23). The difference is that we only use the average pooling layer followed by a convolutional layer and a sigmoid layer, and mix attention (MA) adds these two attentions together to shift the parameters. Because of the highest performance of CA, in a later experiment, we only focused on the performance of MobileNetV2 with or without CA.

### Traditional Data Augmentation

A single flip shift scale is used for the images in the training set. To ensure comparable dataset size, we rotated scar images 8 times and control images 72 times. Finally, 6,684 MI augmentation images and 7,451 control augmentation images were generated. These augmented images formed an image pool, which was sampled randomly in different TA scales. In these experiments, we used 4x augmentation. We first sampled 453 control images to match the MI image size of 557. Upon sampling randomly from the image pool, we generated 2,228 MI and 2,228 control images as the training dataset.

### GAN Augmentation

PGGAN, a generative adversarial network (GAN) variant, has greater power in generating 1,024 × 1,024 high-resolution image platform from a random noise (24). Different from the traditional GAN training process, PGGAN trains with progressive growth resolution. The training starts with a low spatial resolution of 4 × 4 pixels. As the training advances, the generator and discriminator layers increase to match the spatial image resolutions, and hyperparameter α continues to update for a smooth transition to a higher resolution. In our experiments, we generated 256 × 256 scar and control MR images. The overview of PGGAN architecture is shown in Supplementary Figure 4. To quantitatively analyze the shift effect to PGGAN, ImageNet pretrained MobileNetV2 is used again to calculate Frechet inception distance (FID) in equation 1:


(1) $$\begin{array}{r} FID={∥\mu_{r}-\mu_{g}∥}^{2}+Tr\left(\underset{r}{\sum }+\underset{g}{\sum }-2{\left(\underset{r}{\sum }*\underset{g}{\sum }\right)}^{\frac{1}{2}}\right) \end{array}$$

where μ_*r*_, μ_*g*_ are feature mean of real images and generated images, and ∑_r_, ∑_g_, are feature covariance matrix of real images and generated images. Low FID means a low distance from real image distribution to generated image distribution.

Different from PGGAN, CycleGAN generates a fake image from the real image. In CycleGAN, two-cycle consistency losses are introduced to enforce the generated image (G) and reconstructed image (F) from G to be consistent with each other (25). Since CycleGAN uses actual images as an input, we expect the stable generated images to have better classification accuracy with CycleGAN. Because GAN needs a large dataset for training, TA image pool is used to train a GAN. For better GAN training results, we generate images from each category separately.

### Filtration of the Unusual-Looking Images

Principal component analysis (PCA) is a typical technique to reduce data dimensions and visualize data structure. K-means is a classical unsupervised algorithm to cluster data by calculating individual sample distance. We used MobileNetV2 pretraining by ImageNet to extract 1000-dimension features of each image. Next, we used PCA to map the 1000-dimension embedding features into one-dimensional latent space. We assumed that nearly 30% of the values, one standard deviation σ away from the mean, could be considered as unusual images (outliers), which were filtered out from the algorithm.

### Gradient-Weighted Class Activation Mapping

Because the last convolutional layers contain detailed spatial information, Grad-CAM uses the gradient information flowing into the last convolutional layer of the CNN to look for semantic class-specific information in the images (26). We generated a heat map of all the test images by this method to visualize the region of interest for CNN and implement the quantitative analysis.

### Results

#### Cardiac MRI Showed Anterior Wall Thinning and Scarring in Mice With Coronary Artery Ligation

**Figure 1:**
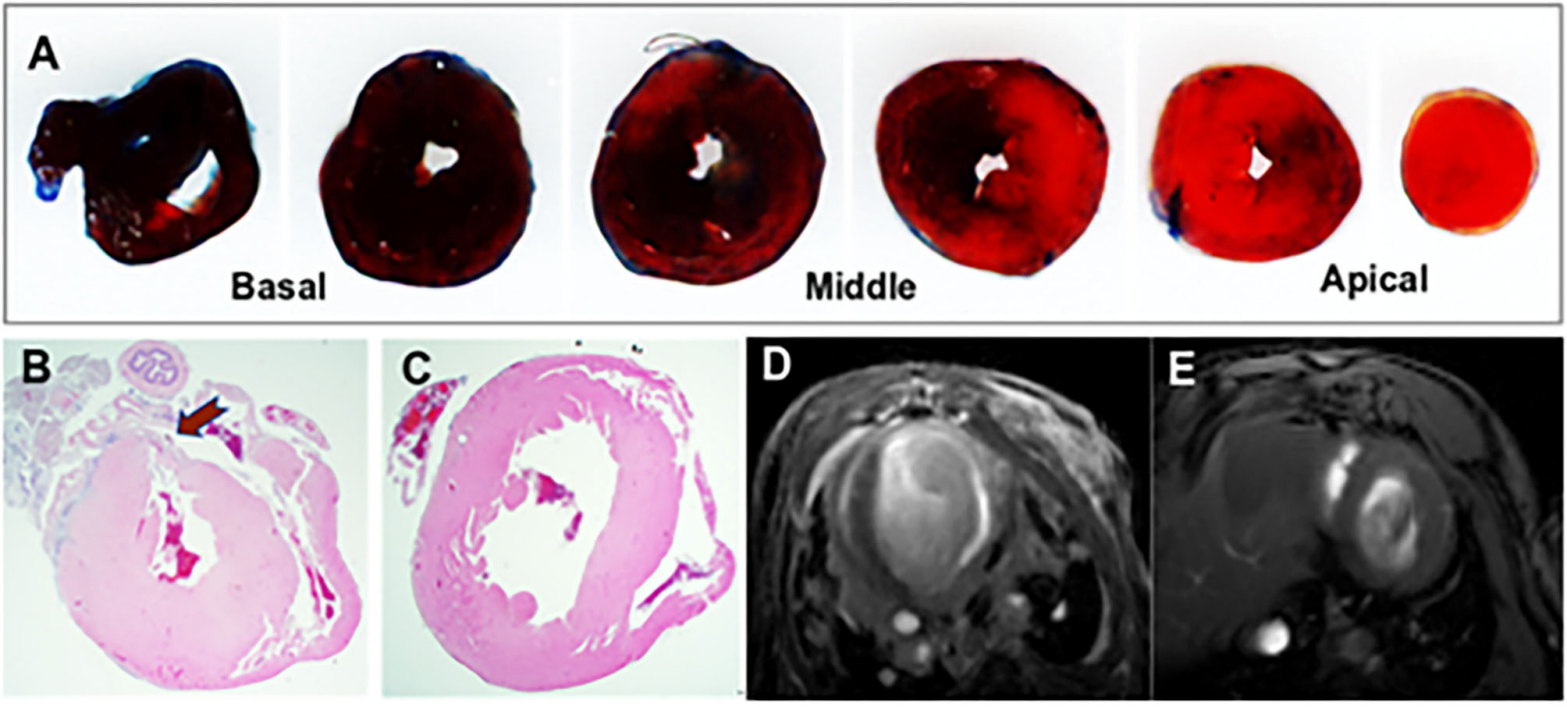
Representative images from the mouse model of acute myocardial infarction (MI). **(A)** TTC stained mouse heart sections at 1 mm after myocardial infraction. The viable myocardium appeared in blue and the area at risk (AAR) in bright red. **(B)** Histological demonstration of a thinned myocardium (shown by an arrow) in a mouse that underwent left anterior descending artery ligation. **(C)** Comparative histology from a normal control mouse. **(D)** Contrast-enhanced cardiac MRI showing anterior wall scar and wall thinning in a mouse model. **(E)** Cardiac MRI showing normal left ventricular morphology.

Acute MI led to regional changes in myocardial morphology as demonstrated by histology and cardiac MRI. Evans Blue/TTC method was employed to assess infarct size as shown in Figure 1A. The severity of myocardial injury following LAD ligation was calculated as the ratio of the area at risk (AAR) to left ventricular area (LV) (AAR/LV: 32.18% ± 13.32, *n* = 6), assessed across the heart at 1 mm intervals from base to apex. Representative images illustrating the TTC-based histological confirmation of myocardial infarction, and an abnormal gadolinium enhancement on contrast-enhanced cardiac MRI are shown in Figure 1. Most of the anterior wall contained the myocardial scar. The remaining regions that had no scar were considered viable remote regions.

### The Principal Component Analysis Showed a Gaussian Distribution of the Data Dimensions

**Figure 2:**
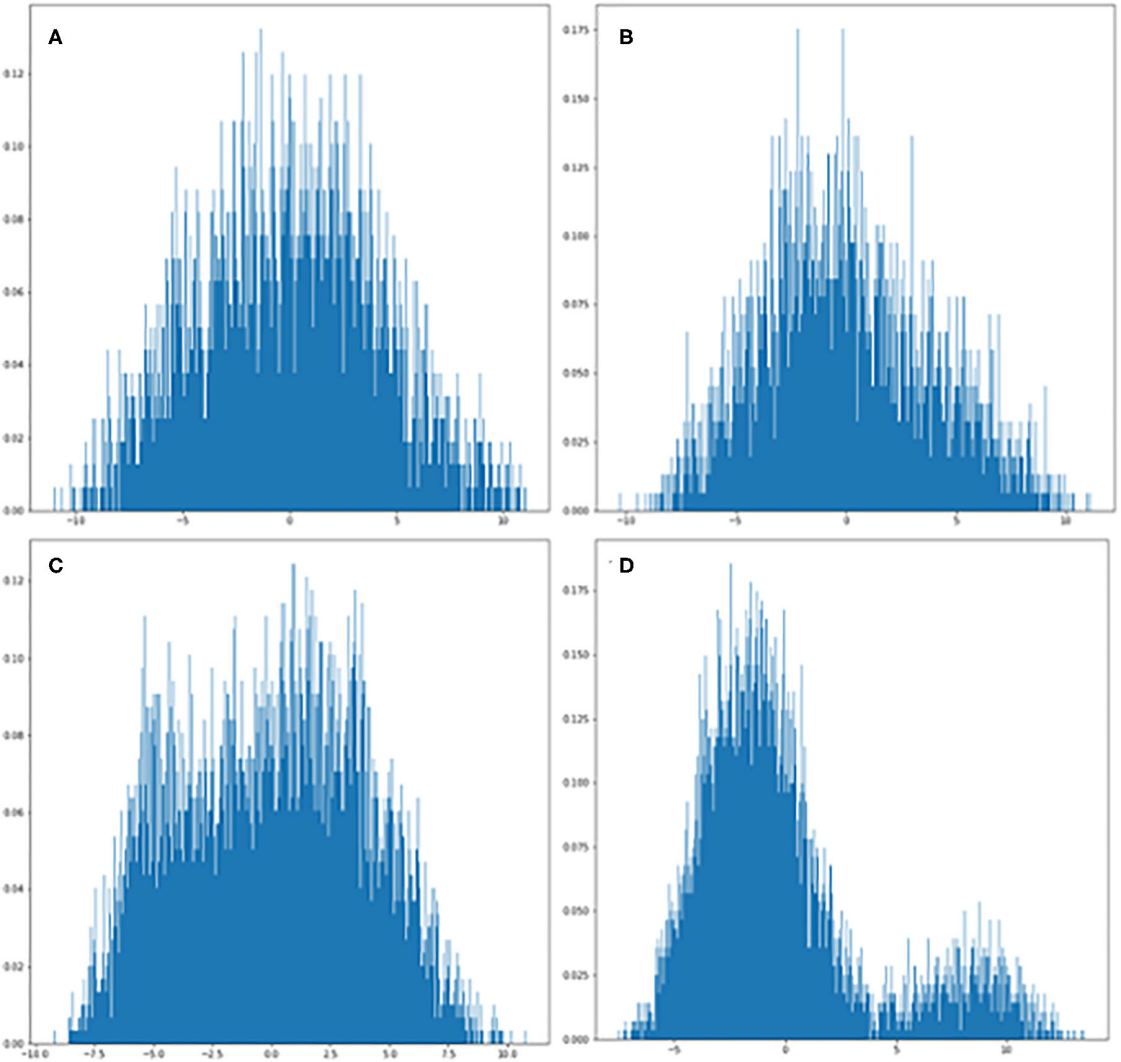
PCA plots of GAN-generated images from each category. **(A)** Visualization of ischemic scar image set generated from PGGAN. **(B)** Visualization of control image set generated from PGGAN. **(C)** Visualization of ischemic scar image set generated from CycleGAN. **(D)** Visualization of control image set generated from CycleGAN.

We used the PCA method to reduce the dimensionality of large data sets by transforming data variables into a smaller one, but preserving most of the information of the larger set. The PCA analysis of feature distribution is shown in Figure 2. Figures 2A,B show the normal distribution of the features generated from PGGAN. Therefore, we used k-means of one to calculate the Euclidean metric between each data point with a mean. Figures 2C,D demonstrate that the data features are subjected to binary-variate Gaussian distribution. This algorithm enabled us easier data exploration, and thus making data analysis much easier and faster for k-means without extraneous variables to process.

### MobileNetV2 With Heatmap Generation Had 100% Accuracy to Classify Acute MI in Mice

**Figure 3:**
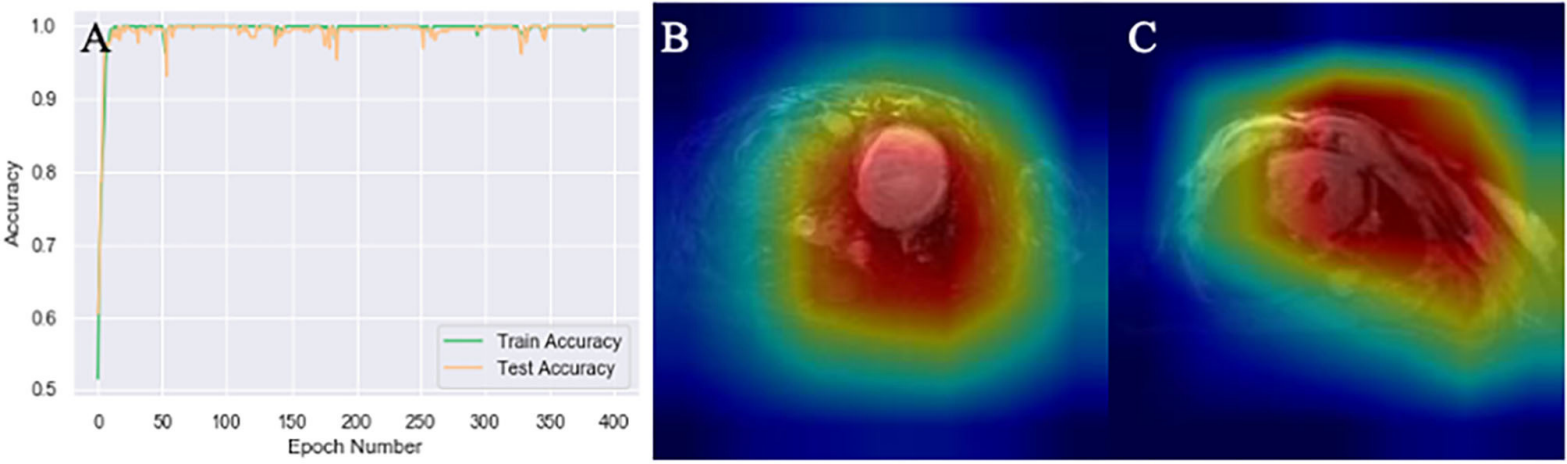
Mouse MRI heatmaps and training curves generated by MobileNetV2. **(A)** Classification accuracy curve. The test accuracy was at 100% after several epochs that stabilized the model. **(B,C)** Heatmaps generated by Gcam from acute myocardial infarction (MI) and control mice, respectively.

After multiple epochs in the training dataset with a stabilized training model, we generated the learning curves to determine the suitability fit to the training dataset. The MobileNetV2 classification accuracy curve demonstrated 100% accuracy in mice as illustrated in Figure 3. Because the accuracy was so high, further data augmentation algorithm was not applied. The heatmap of MobileNetV2 was focused on the whole thoracic cavity, which also includes the ventricles with acute MI.

### Compared to the Spatial or Mixed Attention Modules, the Channel Attention Module Had the Highest Accuracy

**Figure 4:**
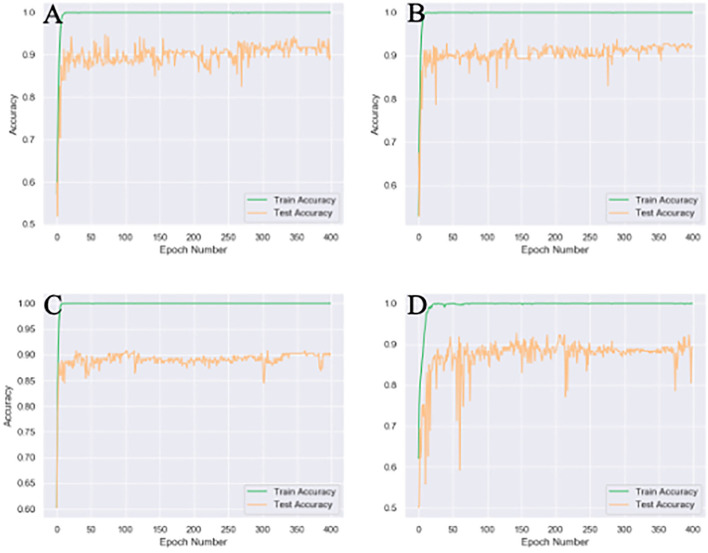
Different attention unit MobileNetV2 training results of 4x traditional augmentation. **(A)** Spatial attention (SA) accuracy curve (89.8% testset accuracy). **(B)** Channel attention (CA) accuracy curve (92.2% testset accuracy). **(C)** Accuracy curve without any attention (89.8% testset accuracy). **(D)**, Mix attention (MA) accuracy curve (89.3% testset accuracy). The accuracy curve fluctuate more violently without any attention module and the final accuracy is the lowest.

Different attention modules were tested based on 4x traditional augmentation. Compared to spatial or mixed attention training modules, CA showed the highest accuracy with the most stable system, as illustrated in Figure 4. The combined module might have been less efficient than the original CA, since the summation of the attention modules in different axes could partly counterbalance the original features.

### PGGAN Shifted by K-Means Removed the Data Outliers, CycleGAN Improved the Data Outline

**Figure 5:**
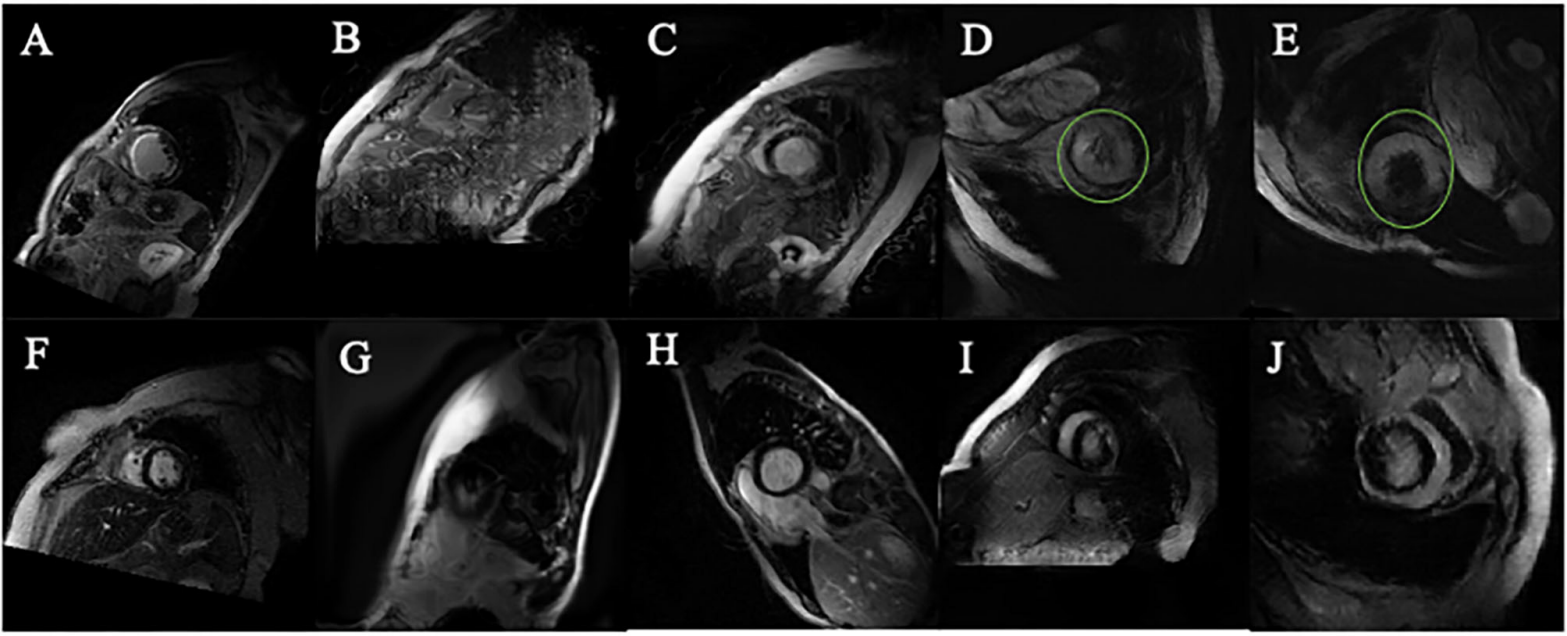
Images generated by PGGAN and CycleGAN. **(A)** MI image from original data. **(B)** MI image generated form PGGAN. **(C)** MI image shifted by kmeans after PGGAN generation. **(D)** MI image generated from CycleGAN. **(E)** MI image shifted by kmeans after CycleGAN generation. **(F)** Control image from original data. **(G)** Control image generated form PGGAN. **(H)** Control image image shifted by kmeans after PGGAN generation. **(I)** Control image generated from CycleGAN. **(J)** Control image shifted by kmeans after CycleGAN generation. Green circle in **(D,E)** is manually added so as to mark the abnormal morphology of the generated image.

Compared to the original images (Figures 5A,F), Many PGGAN generated images are of unusual shape as demonstrated in Figures 5B,G. Since the original training dataset is relatively small, a small raw dataset does not provide sufficiently high variance information for stable image generation. Table 1 shows that k-means selection reduces FID, and therefore should be an effective method to remove unusually shaped images. Some typical PGGAN generated images with k-means are shown in Figures 5C,H. Although some images are still of unusual shape, k-means filters the outliers and reduces the number of unusually shaped images.

**Table 1:** Generation of FID score from PGGAN.

	**FID**
PGGAN (MI)	289.208
PGGAN + kmeans (MI)	285.746
PGGAN (control)	323.129
PGGAN + kmeans (control)	322.873

*PGGAN, the FID score of PGGAN; PGGAN+kmeans, FID of kmeans sift of original PGGAN generation; MI, myocardial infarction; Control, normal images from the control group*.

Figures 5D–J show CycleGAN generated images. CycleGAN translates images from the real images in different source domains. Unlike the images generated by PGGAN, all CycleGAN-generated images represent a clearer tissue outline. The main problem associated with CycleGAN is that scar tissue images are imperfectly translated from the normal image domain. In our study, this resulted in the overestimation of the scar size (green circle, Figures 5D,E) with a hollow (black) core. This semantic difference would be hard to be shifted by k-means. The classification results also showed that k-means is more effective for PGGAN generation shift than for CycleGAN generation shift.

### Combination of GAN Augmentation and Kmeans Selection Was a Reliable Method to Improve CNN Performance

All classification results of different augmentation methods are presented in Table 2. The performance accuracy (high-to-low) is determined to be PGGAN augmentation, TA and CycleGAN. In particular, 4x PGGAN-k-means based augmentation shows the best accuracy (92.7%). We infer that perhaps the inferior performance of CycleGAN was due to unthorough translation from source image domain. The GAN augmentation classification results without k-means are shown in Table 3. Taken together, k-means selection enhances PGGAN augmentation accuracy removing the unusually-shaped images. Images generated by CycleGAN have a better edge definition. Additionally, the ROC (receiver operating characteristic) curves are shown in Supplementary Figure 5.

**Table 2:** Comparative analysis of various classification approaches.

	**Acc (%)**
MobilenetV2 (raw)	55.8
MobilenetV2 (raw) (CA)	50.5
TA base MobilenetV2	83.5
TA base MobilenetV2 (CA)	88.8
4x TA MobilenetV2	90.3
4x TA MobilenetV2 (CA)	92.2
PGGAN+kmeans base MobilenetV2	84.5
PGGAN+kmeans base MobilenetV2 (CA)	88.3
4x PGGAN+kmeans MobilenetV2	89.8
4x PGGAN+kmeans MobilenetV2 (CA)	**92.7**
CycleGAN+kmeans base MobilenetV2	83.5
CycleGAN+kmeans base MobilenetV2 (CA)	88.3
4x CycleGAN+kmeans MobilenetV2	87.4
4x CycleGAN+kmeans MobilenetV2 (CA)	91.7

*Acc (accuracy), kmeans (k = 1, removes PGGAN generative images. K = 2, removes CycleGAN generative images), base (augmentation applied to increase normal set size same as acute set size so as to mitigate the imbalance). TA, traditional augmentation; CA, channel attention. Bold means highest accuracy*.

**Table 3:** GAN augmentation classification results without kmeans.

	**Acc (%)**
4x PGGAN MobilenetV2	81.1
4x PGGAN MobilenetV2 (CA)	87.9
4x CycleGAN MobilenetV2	82.5
4x CycleGAN MobilenetV2 (CA)	90.7

### Higher Accuracy Was Correlated With a Smaller Region of Interest

**Figure 6:**
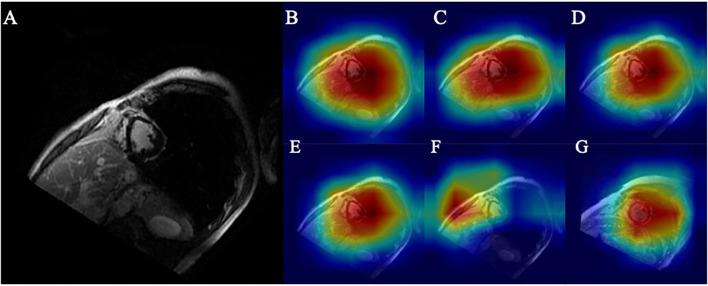
**(A)** Original ischemic scar image. **(B)** Heatmap of base model. **(C)** Heatmap of CA embedded to base model. **(D)** Heatmap of CA embedded to base model with 4x traditional augmentation. **(E)** Heatmap of CA embedded to base model with 4x PGGAN augmentation. **(F)** Heatmap of CA embedded to base model with 4x CycleGAN augmentation. **(G)** Heatmap of CA embedded to base model with 4x traditional augmentation of image in the control group.

As shown in Figures 6D,G, the myocardial scar was correctly localized through Grad-CAM. The ROI (marked red) represents the scar-bearing myocardial segment. However, Grad-CAM was not sensitive enough to locate the myocardial scar at the border zones. One possible explanation for this caveat could be that without pixel-level labeling of myocardial scar, the self-localization of CNN from a small Grad-CAM training set is limited. Further augmentation results with smaller ROI and higher accuracy as shown in Figures 6A–F and Table 2.

Higher accuracy is likely derived from a precise location of the ROI. A similar heatmap generated from TA (Figure 6D) and from PGGAN augmentation (Figure 6E) reveals that ROI generated from PGGAN+k-means is the same as in TA, and the classification accuracies from PGGAN+k-means augmentation (92.7) and TA (92.2) are similar. This also suggests that attention shift could be the reason for inferior training results from CycleGAN (91.7). Since CycleGAN (Figure 6F) provides higher emphasis to the subdominant features around the ventricles, this can lead to attention shift.

## Discussion

Despite recent advances in the field of deep learning to predict cardiovascular outcomes (27, 28), there are limited data examining the accuracy of myocardial scar classification. This is the first multidisciplinary study that combines the preclinical and clinical approaches to develop a tissue-validated classification and augmentation algorithms in subjects with an ischemic myocardial scar. Our CNN model introduces an attentional block-based data processing approach to improve MobileNetV2 classification of myocardial scar. We also report that GAN is an effective method to mitigate the data imbalance and present a comparative data analysis algorithm to show which GAN-type is must suitable to augment myocardial scar imaging. Finally, we combine k-means and PCA to identify abnormal images with the goal of improving the augmentation effects in advance.

Quantitative interpretation of myocardial scar has remained a challenging task despite the use of automated edge-detection techniques (29, 30). The manual segmentation is time-consuming and subjective, which can lead to high intra- and inter-observer variations. To date, there were no studies that attempted the gold standard approaches of myocardial scar classification using a histological validation in an acute MI model, and clinical validation in patients with chronic ischemic myocardial remodeling.

Our initial data classification algorithm tested in a mouse model of acute MI demonstrated 100% accuracy for the classification of acute MI. Although this approach was highly promising and did not require additional data filtration or augmentation algorithms, there can be limitations of fully extrapolating the mouse data into the clinical scenario. First, the mouse model of acute MI was developed by irreversible occlusion of the left anterior descending coronary artery, which is not entirely representative of our patient population with chronic CAD. Second, the mouse model of acute MI was studied within the first two weeks of MI induction. Nevertheless, mice MRI data showed smaller variance and higher interpretation accuracy compared to the human data. This preclinical training model provided additional intimations to advance classification performance and improve accuracy.

Generally, our results show the classification accuracy obtained from combined PGGAN and k-means is comparable to traditional data augmentation. The inferior data augmentation from CycleGAN is likely due to unsupervised image-to-image translation generated without prior restriction. For image-to-image augmentation, training from more powerful translation GAN like UGATIT platforms may enhance performance and improve accuracy (31). As for noise-to-image augmentation, newer approaches with layer normalization instead of pixel norm in PGGAN could be attempted to improve the semantic understanding.

Although channel attention has shown the highest model stability in our experiments, other attention modules could also be utilized to fulfill additional tasks. Powerful spatial attention modules such as the non-local layer or graph reasoning layer could be optimized and then applied to our system for the accurate classification of myocardial scar in different coronary artery territories (32, 33). Since the image-shifting is a commonly encountered challenge, newer outlier detection algorithms could be utilized to shift the generated images more effectively. The unusual-looking images pose a semantic difference with well-looking images. In particular, for CycleGAN generated images, an unthorough translation of the myocardial scar leads to a high-level of semantic difference.

In contrast with CycleGAN-based image generation, k-means outlier selection is based on the Gaussian distribution rule. Although the feature extraction step from the pretrained MobileNetV2 involves image semantic information, this has a limited utility for our imaging processing. This advantage of PGGAN over the CycleGAN could be the reason for the higher accuracy of PGGAN data augmentation after k-means selection. Our data suggest that the use of an algorithm with an integrated medical image semantic extraction module can extract outliers, and enhance classification performance.

## Limitations

Our study has a few limitations which can be overcome with future research. This study has a small image dataset size as described above. However, we have, at least in part, addressed this issue by rotating the scar images 8 times and control images 72 times, followed by scaling, shifting and flipping images one time, so that ~7,000 images with balanced augmentation were obtained. In addition, this study was not designed to study the in-depth mechanisms of ischemic remodeling in mice. Since the image classification was our main goal, the clinical data were not tailored to study the long-term cardiovascular outcomes. Nevertheless, this is the first step toward the training a validation of the myocardial scar classification algorithm using a multidisciplinary approach.

## Conclusions and Future Implications

We have shown that the channel attention is the most effective attention unit to improve CNN performance. We conclude that the data performance can be improved by utilizing a min-max contrast between the discriminator and generator models of GAN. K-means has a strong ability to remove unusually-shaped generated images and thus amplifying the accuracy training from PGGAN augmentation (17).

In the future, besides image interpretation, this model could be applied in several medical applications, including a GAN-based data augmentation anonymization tool for large-scale data sharing, and a clinical training tool to educate medical practitioners. One promising approach is to use this method to augment the size and variability of myocardial scar, which can predict clinical outcomes, including heart failure and sudden arrhythmic events.

## Data Availability Statement

The original contributions presented in the study are included in the article/Supplementary Material, further inquiries can be directed to the corresponding author/s.

## Ethics Statement

The studies involving human participants were reviewed and approved by Human experimental protocols were approved by a institutional review board (IRB) committee from University at Buffalo and all methods involving human/human data were performed in accordance with the relevant guidelines and regulations. Written informed consent for participation was not required for this study in accordance with the national legislation and the institutional requirements. The animal study was reviewed and approved by all preclinical procedures and protocols conformed to institutional guidelines for the care and use of animals in research and were reviewed and approved by the University at Buffalo Institutional Animal Care and Use Committee (IACUC).

## Author Contributions

US supervised the clinical aspects of this study and contributed to the manuscript writing. KZ performed experiments, analyzed data, and contributed to the manuscript writing. BK contributed to the manuscript writing. JL, KM, and SS contributed to pre-clinical experiments and data analysis. LY was responsible for the overall supervision of machine learning techniques and critiqued the manuscript. All authors contributed to the article and approved the submitted version.

## Funding

This research was supported by the National Center for Advancing Translational Sciences of the National Institutes of Health (UL1TR001412) to the University at Buffalo. US received support from NIH/NHLBI (K08HL131987 and R01HL152090) and JL received support from VA/United States (IK2 BX004097/BX/BLRD).

## Conflict of Interest

The authors declare that the research was conducted in the absence of any commercial or financial relationships that could be construed as a potential conflict of interest.

## Publisher's Note

All claims expressed in this article are solely those of the authors and do not necessarily represent those of their affiliated organizations, or those of the publisher, the editors and the reviewers. Any product that may be evaluated in this article, or claim that may be made by its manufacturer, is not guaranteed or endorsed by the publisher.
