# Supplementary material for: Modified GAN Augmentation Algorithms for the MRI-Classification of Myocardial Scar Tissue in Ischemic Cardiomyopathy
Source: Front Cardiovasc Med. 2021 Sep 13;8:726943. doi: 10.3389/fcvm.2021.726943 (PMC8473636; doi:10.3389/fcvm.2021.726943)
Supplement: Supplementary file 1 [file Presentation_1.PPTX]

## Slide 1
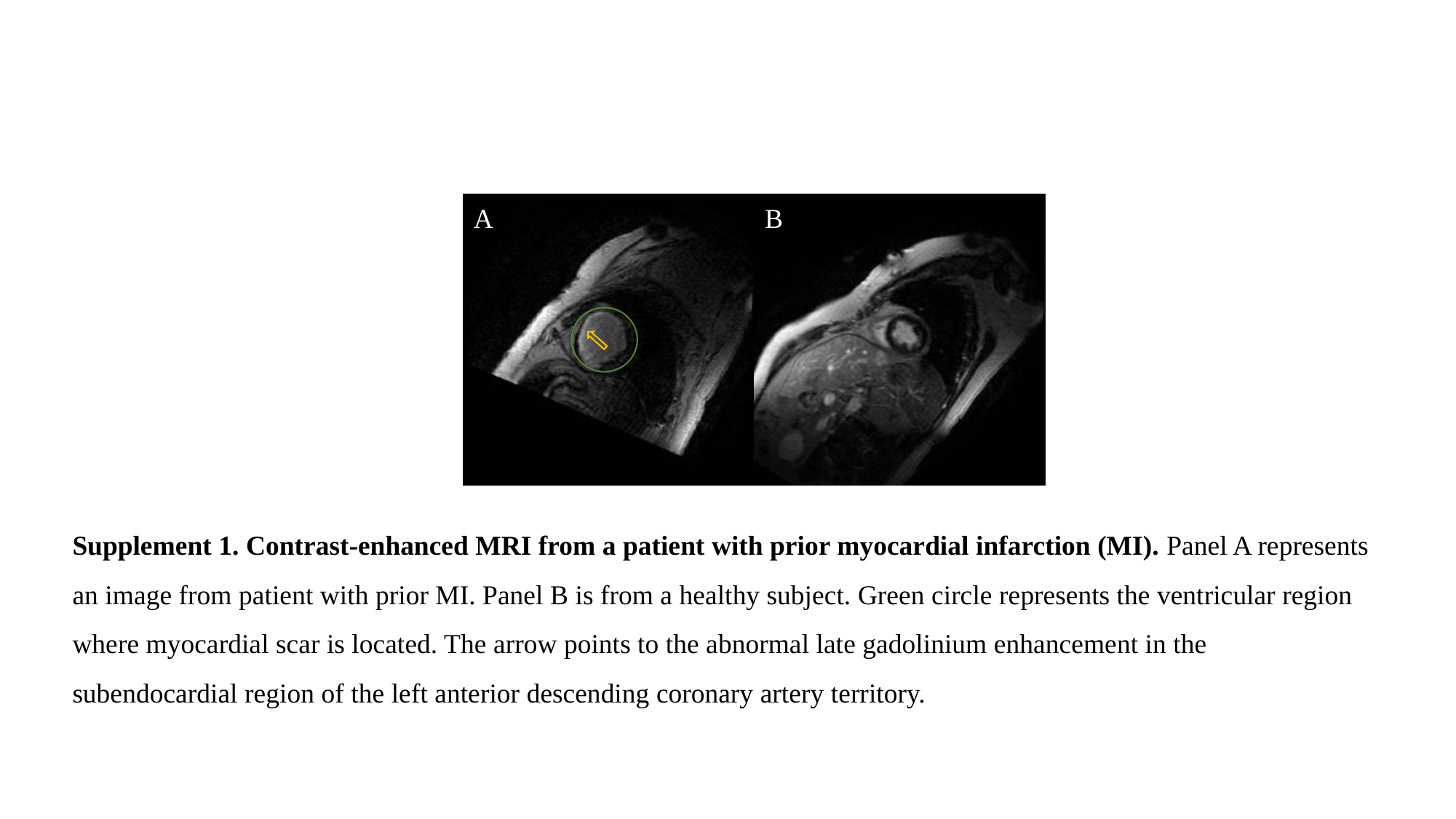

A
B
Supplement 1. Contrast-enhanced MRI from a patient with prior myocardial infarction (MI). Panel A represents an image from patient with prior MI. Panel B is from a healthy subject. Green circle represents the ventricular region where myocardial scar is located. The arrow points to the abnormal late gadolinium enhancement in the subendocardial region of the left anterior descending coronary artery territory.

## Slide 2
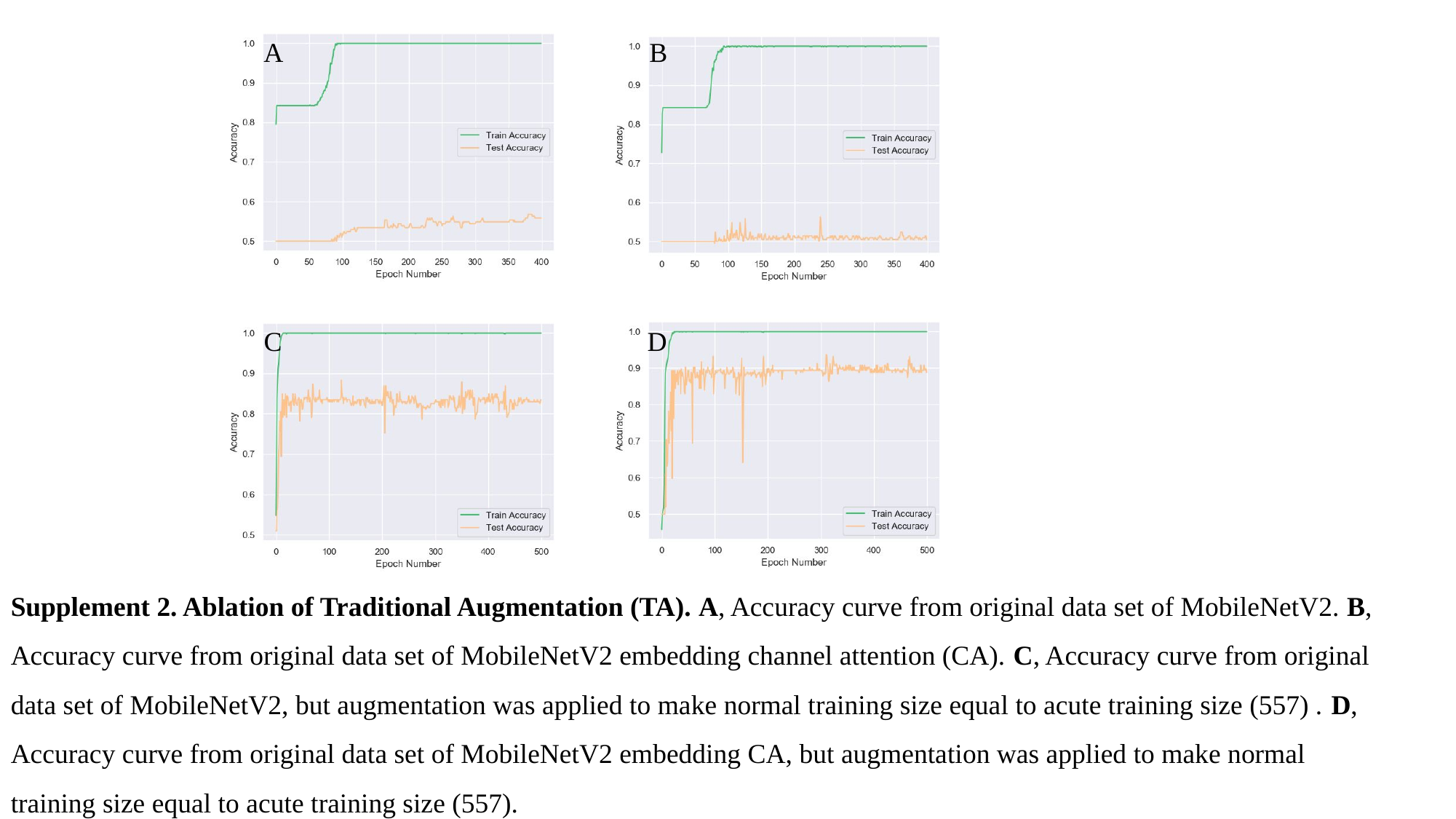

A
B
C
D
Supplement 2. Ablation of Traditional Augmentation (TA). A, Accuracy curve from original data set of MobileNetV2. B, Accuracy curve from original data set of MobileNetV2 embedding channel attention (CA). C, Accuracy curve from original data set of MobileNetV2, but augmentation was applied to make normal training size equal to acute training size (557) . D, Accuracy curve from original data set of MobileNetV2 embedding CA, but augmentation was applied to make normal training size equal to acute training size (557).

## Slide 3
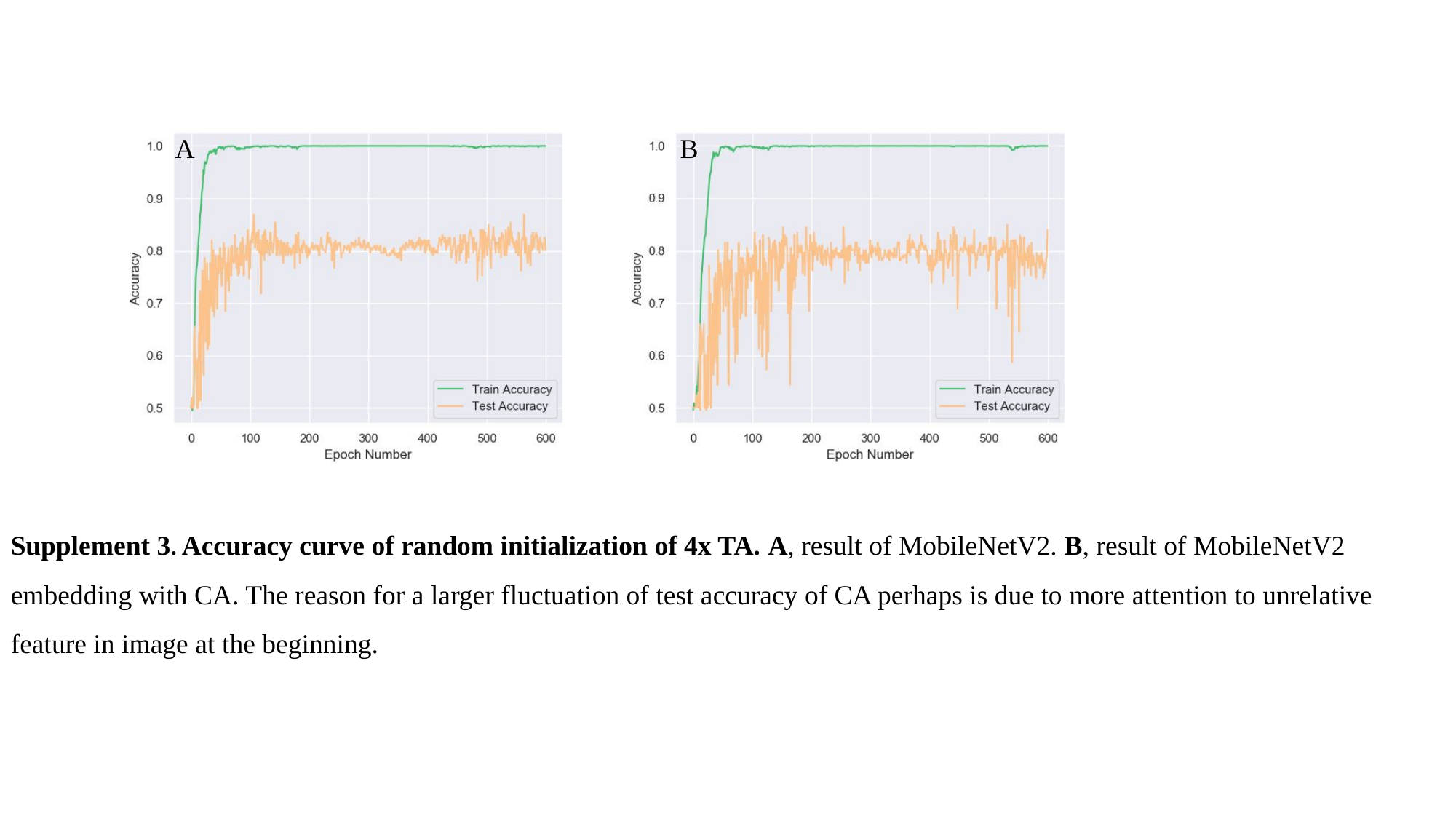

A
B
Supplement 3. Accuracy curve of random initialization of 4x TA. A, result of MobileNetV2. B, result of MobileNetV2 embedding with CA. The reason for a larger fluctuation of test accuracy of CA perhaps is due to more attention to unrelative feature in image at the beginning.

## Slide 4
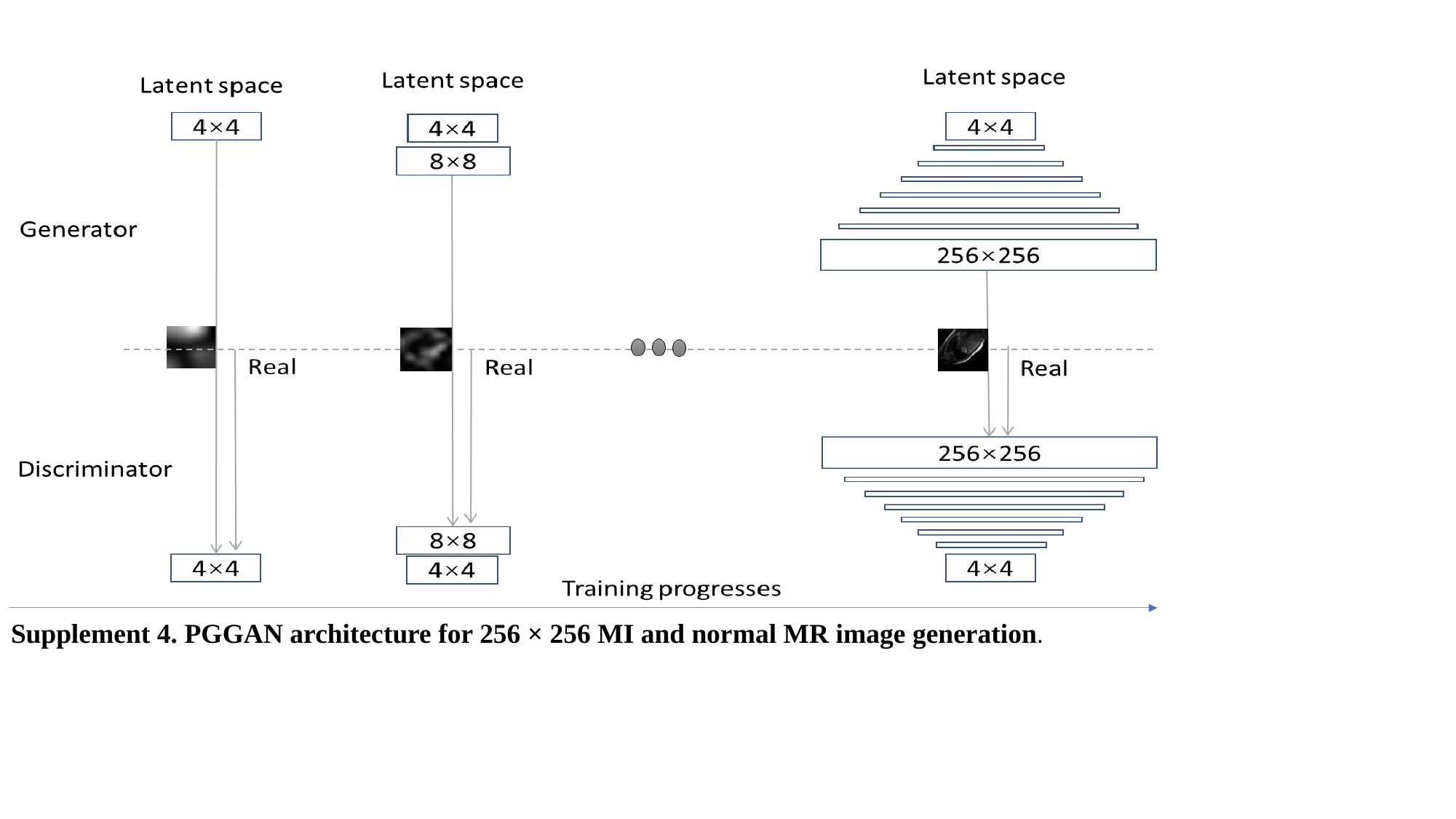

Supplement 4. PGGAN architecture for 256 × 256 MI and normal MR image generation.

## Slide 5
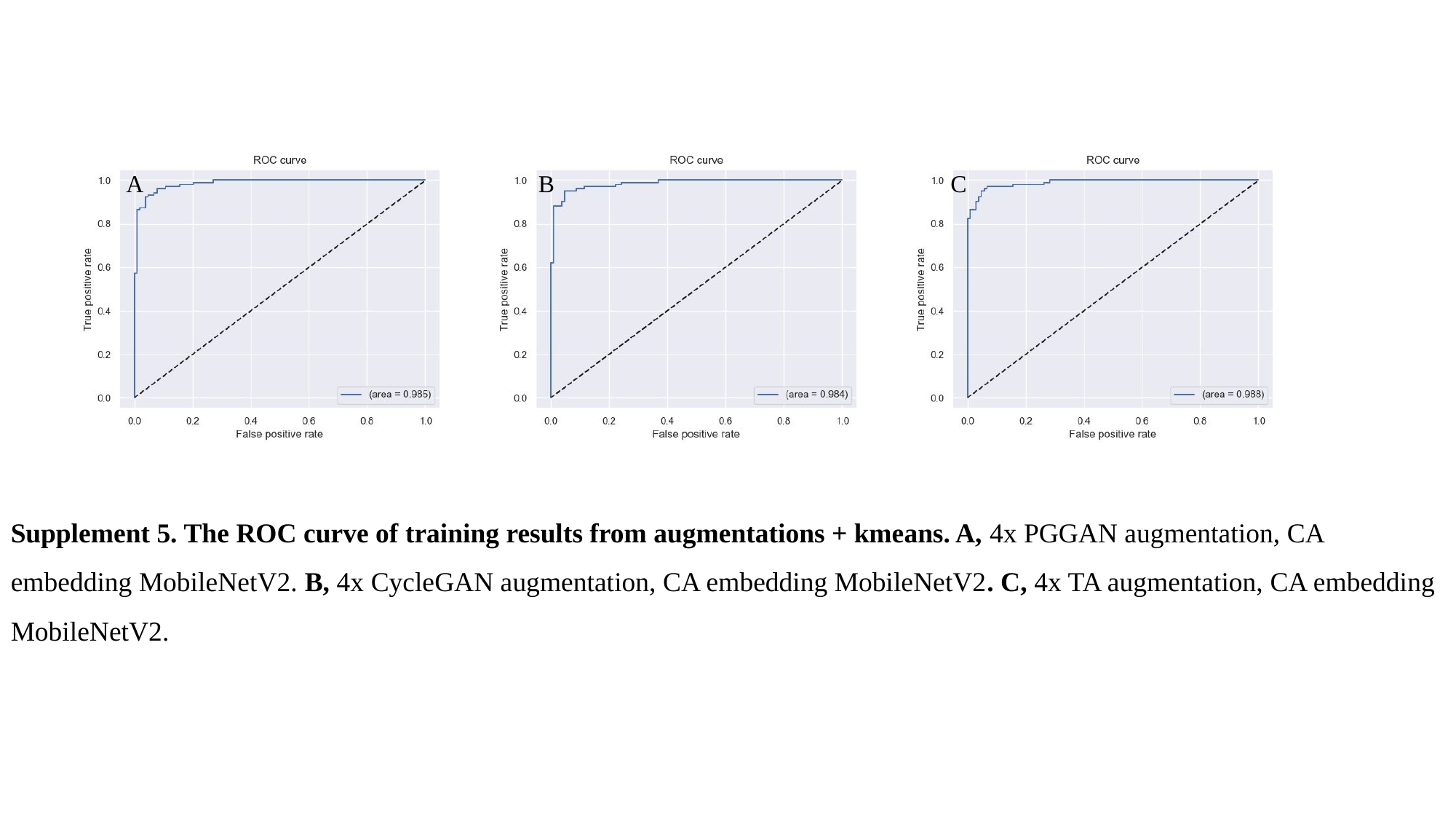

A B C
Supplement 5. The ROC curve of training results from augmentations + kmeans. A, 4x PGGAN augmentation, CA embedding MobileNetV2. B, 4x CycleGAN augmentation, CA embedding MobileNetV2. C, 4x TA augmentation, CA embedding MobileNetV2.
